# Thicknesses of the retina and choroid in children with retinitis pigmentosa

**DOI:** 10.1186/s12886-023-02772-0

**Published:** 2023-01-17

**Authors:** Cheng Li, Chunxia Peng, Chengyue Zhang, Ningdong Li

**Affiliations:** Department of Ophthalmology, Beijing Children’s Hospital, Capital Medical University, National Center for Children’s Health, No.56 Nanlishi Road, Xicheng District, Beijing, 100045 China

**Keywords:** Retinitis pigmentosa, Retinal and choroidal thicknesses, XLRP and ADRP

## Abstract

**Purpose:**

To compare the retinal thicknesses (RT) and choroidal thicknesses (CT) in retinitis pigmentosa (RP) children with those of healthy children using enhanced depth imaging (EDI) optical coherence tomography (OCT). The RT and CT in different genetic subgroups of autosomal dominant RP (ADRP) and X-linked inheritance RP (XLRP) were further studied to investigate the characteristics of retinal and choroidal changes in the early stages of RP.

**Method:**

A retrospective analysis was performed on a group of patients with RP who underwent EDI-OCT. Thirty-two children (64 eyes) with RP and 28 age- and refraction-matched healthy children (56 eyes) were included in the study. Seven of the 32 RP children (14 eyes) had X-linked inheritance RP, and 10 (20 eyes) had autosomal dominant inheritance RP. RT and CT were measured by optical coherence tomography and compared between the 32 children with RP and 28 controls and between 7 XLRP and 10 ADRP children.

**Result:**

Among the 32 children with RP, there were 18 males and 14 females with an average age of 6.6 ± 2.4 years. The mean RT was smaller in the RP group than in the control group at all of the locations. The mean temporal CT was smaller in the RP group (243.76 ± 60.82 μm) than in the control group (275.23 ± 40.92 μm) (*P* = 0.001), while there was no significant thinning on the foveal or nasal side.

The best-corrected visual acuity of the XLRP group (0.40 ± 0.19) was worse than that of the ADRP group (0.68 ± 0.21) (*P* = 0.001), but the disease duration was the same (*P* = 0.685). The mean foveal RT was smaller in the XLRP group (173.85 ± 22.87 μm) than in the ADRP group (192.20 ± 9.70 μm) (*P* = 0.003), while there was no significant thinning at the other locations we studied. The mean temporal CT was smaller in the XLRP group (211.21 ± 69.41 μm) than in the ADRP group (274.45 ± 57.91 μm) (*P* = 0.007); CT measurements in XLRP children showed a more severe reduction on the temporal side.

**Conclusion:**

The choroid in RP children was preferentially smaller on the temporal side of the macula, and retinal thinning was relatively extensive. Children with RP have strong clinical and genetic heterogeneity. The XLRP children demonstrated greater RT reduction at the fovea and greater CT reduction at the temporal side of the macula than the ADRP children. Our findings also provide evidence that the changes in thicknesses may be indicative of the greater severity of XLRP versus ADRP in the early stage.

## Introduction

Retinitis pigmentosa (RP) is an inheritable retinal disorder with an estimated prevalence of 1 in 4000 ~ 5000 in the global population [1]. It can be inherited in an autosomal dominant (AD), autosomal recessive (AR), X-linked (XL), or digenic pattern [2]. Night blindness usually presents as an early symptom of RP, followed by a progressive contraction of the peripheral visual field. The patient’s central vision may be preserved in the early stage but will decrease over time, ultimately resulting in complete blindness when cone cell function is lost entirely. Fundus changes are typically characterized as a waxy pallor of the optic disc with narrowing of retinal blood vessels and formation of bone spicule–like pigment deposits at the mid-periphery of the retina. The electrophysiological response of the patient’s retina in an electroretinogram (ERG) may be diminished or absent.

RP has strong clinical and genetic heterogeneity, with diverse inheritance patterns, disease-causing genes and clinical features. The expressivity of the causative genes is variable among patients with different inheritance patterns and different gene mutations. In the early stage of RP, visual impairment is believed to be milder in AD patients, who have a relatively late onset of illness and retain their central vision into adulthood, than in XL patients, who have a more severe disease course, especially for affected males [3].

Although RP is caused by mutations in many genes, the final pathway of pathological change is apoptosis of the photoreceptor cells (PRC) and the retinal pigment epithelium (RPE). The PRC and RPE atrophy due to loss of cells as the disease progresses; this degradation ultimately leads to structural changes in the whole retina. The choroid lies adjacent to the retina and supplies it with abundant oxygen, blood and nutrients to meet the high metabolic demands of the PRC and RPE. When the retina undergoes structural changes, the choroid, which is entirely composed of vascular tissue, may be involved in retinal vessel narrowing. In this study, we evaluated the anatomical changes in the retina and choroid in patients with autosomal dominant RP (ADRP) and X-linked RP (XLRP).

## Materials and methods

### Participants

We conducted a retrospective study including thirty-two children (64 eyes) with RP and 28 age- and refraction-matched healthy children (56 eyes). Patients with XLRP and ADRP who were under 12 years of age were recruited for this study. The diagnosis of RP was based on the following criteria: (1) reduction or extinction of electrophysiological responses recorded in an ERG; and (2) typical fundus changes on retinoscopic examination. Children in the healthy control group had no eye diseases except for some refractive errors. Patients were divided into the XLRP and ADRP groups according to their disease-causing genes. The duration of symptoms and onset age of XLRP and ADRP were matched (shown in Table 1). All participants underwent ophthalmologic examinations, including evaluations of best-corrected visual acuity using Snellen charts, slit-lamp examination of the anterior segment of each eye, and examination of the vitreous and fundus by ophthalmoscopy and photography [4]. Refractive error was examined using a Welch Allyn VS100 apparatus. Full-field ERGs were recorded using the RETI-scan 21 system (Roland company, Germany) according to the standards of the International Society for Clinical Electrophysiology of Vision (http://www.iscev.org).

**Table 1:** Demographic characteristics of the participants

	Control group (*n* = 28, 56 eyes)	RP group (*n* = 32, 64 eyes)	*P* value	RP(*n* = 17 with two gene type)		
				XLRP (*n* = 7, 14 eyes)	ADRP (*n* = 10, 20 eyes)	*P* value
Sex (male/female)	15/13	18/14	*P* = 0.602	5/2	6/4	*P* = 0.627
BCVA	–	–	–	0.40 ± 0.19	0.68 ± 0.21	*P* = 0.001
Age (years)	7.1 ± 2.2	6.6 ± 2.4	*P* = 0.314	5.1 ± 0.89	6.1 ± 2.1	*P* = 0.314
Disease duration (years)	–	–	–	2.57 ± 1.13	2.4 ± 1.07	*P* = 0.685
Spherical equivalent	−1.20 ± 3.65	−1.93 ± 4.88	*P* = 0.303	–	–	–
Genotypes	–	–	–	RP2, RPGR	TOPOR, PRPF8, PRPF31, RHO, HK1, PRPF4, RDH12 RPE65, SEMA4A	–

*Abbreviations*: *XLRP* X-linked RP, *ADRP* Autosomal dominant RP, *BCVA* Best-corrected visual acuity

Patients were excluded if they had myopia of more than 6 dioptres (D), hyperopia higher than 3 D, astigmatism higher than 3 D, cataracts, macular oedema or epiretinal membrane, because these factors might interfere with the accurate measurement of the retinal and choroidal thicknesses [5]. This study complied with the tenets of the Declaration of Helsinki and was approved by the Ethics Committee of the Beijing Children’s Hospital.

### OCT examinations

**Fig. 1:**
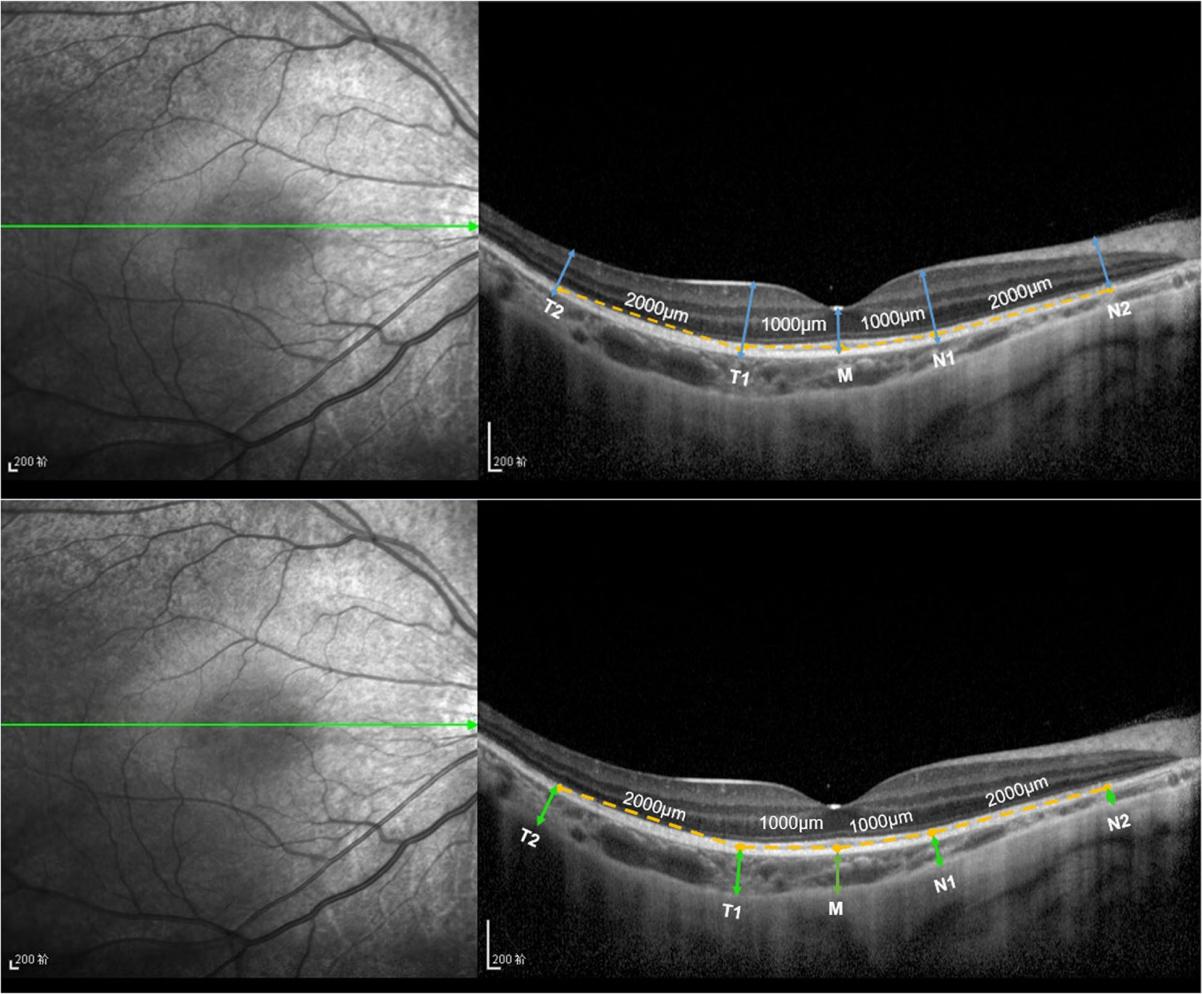
Measurement map of the thicknesses of the retina and choroid. The retinal thickness (RT) is the distance between the inner limiting membrane and the outer border of the RPE; the choroid thickness (CT) is measured from the outer border of the RPE vertically to the inner scleral border. M: foveal thickness; N1: thickness 1000 μm nasal to the fovea; N2: thickness 3000 μm nasal to the fovea; T1: thickness 1000 μm temporal to the fovea; T2: thickness 3000 μm temporal to the fovea

OCT measurements were obtained using a Spectralis OCT apparatus with a wavelength of 870 nm (Heidelberg Engineering GmbH, Germany) in enhanced depth imaging (EDI) acquisition mode. One section through the macular fovea and the optic disc, composed of 100 averaged scans, was obtained. All children were scheduled for examination in the afternoon. A horizontal high-resolution line scan passing through the fovea in primary gaze was chosen for detailed analysis. The thicknesses of the retina and choroid were measured manually using the calliper function in the Heidelberg reader software [6]. The retinal thickness (RT) was measured between the inner limiting membrane and the outer border of the RPE, and thickness measurements were obtained at the fovea and at 1000 μm and 3000 μm nasal and temporal to the centre of the fovea in a horizontal section [7]. The choroid thickness (CT) was measured from the outer border of the RPE vertically to the inner scleral border at the fovea and at 1000 μm and 3000 μm nasal and temporal to the centre of the fovea in a horizontal section (shown in Fig. 1) [8]. Both the RT and CT were measured by the same experienced physician; the measurements were repeated three times and averaged.

### Molecular diagnosis

Three millilitres of peripheral venous blood was collected from the patients and their matched control participants. The patient’s DNA was extracted from the lymphocytes according to a standard protocol (Roche Biochemical, Inc.). Exome sequencing was performed by the commercial Myogenomic Company (Beijing, China). Sequences were analysed using the Seqman program in DNASTAR software (DNASTAR Inc., Madison, WI). The online tools Polyphen-2 and SIFT were used to predict the possible impact on the structure and function of a protein after an amino acid substitution. Mutations were named following the nomenclature recommended by the Human Genomic Variation Society (HGVS). According to the ACMG criteria, the gene variants in our study were pathogenic.

### Statistics

The retinal and choroidal thicknesses were compared between different groups. Statistical analysis was performed using a statistical software package (SPSS, version 23.0; IBM, Armonk, NY, USA). Frequency and descriptive statistics were used for data analysis. Demographic and clinical characteristics, including age, CT and RT, were compared using independent sample t tests. All the data were normally distributed, and sex was compared using the chi-squared test. Differences were considered significant if *p* < 0.05.

## Results

**Fig. 2:**
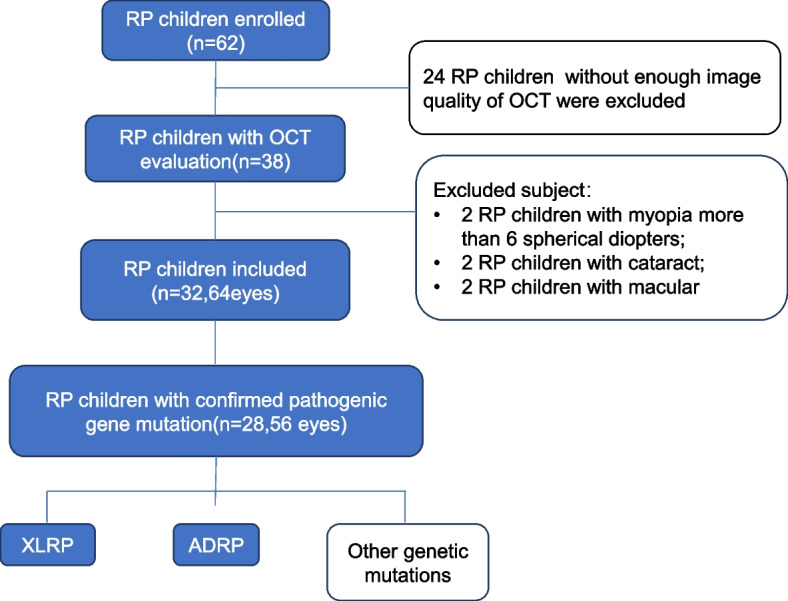
Flowchart of the paediatric participants with RP.(Abbreviations: RP: retinitis pigmentosa; OCT: optical coherence tomography; XLRP: X-linked inheritance RP; ADRP: autosomal dominant inheritance RP)

Sixty-four eyes of 32 children with RP (18 males and 14 females) and 56 eyes of 28 healthy controls (15 males and 13 females) were included in the study. A flowchart of patient recruitment is shown in Fig. 2. There was no significant difference in age, sex, or spherical equivalent between the RP and control groups (shown in Table 1). Fourteen eyes of 7 children with X-linked inheritance (5 males and 2 females) and 20 eyes of 10 children with autosomal dominant inheritance (6 males and 4 females) were included in the study and allocated to the XLRP and ADRP groups, respectively (Table 1).

**Fig. 3:**
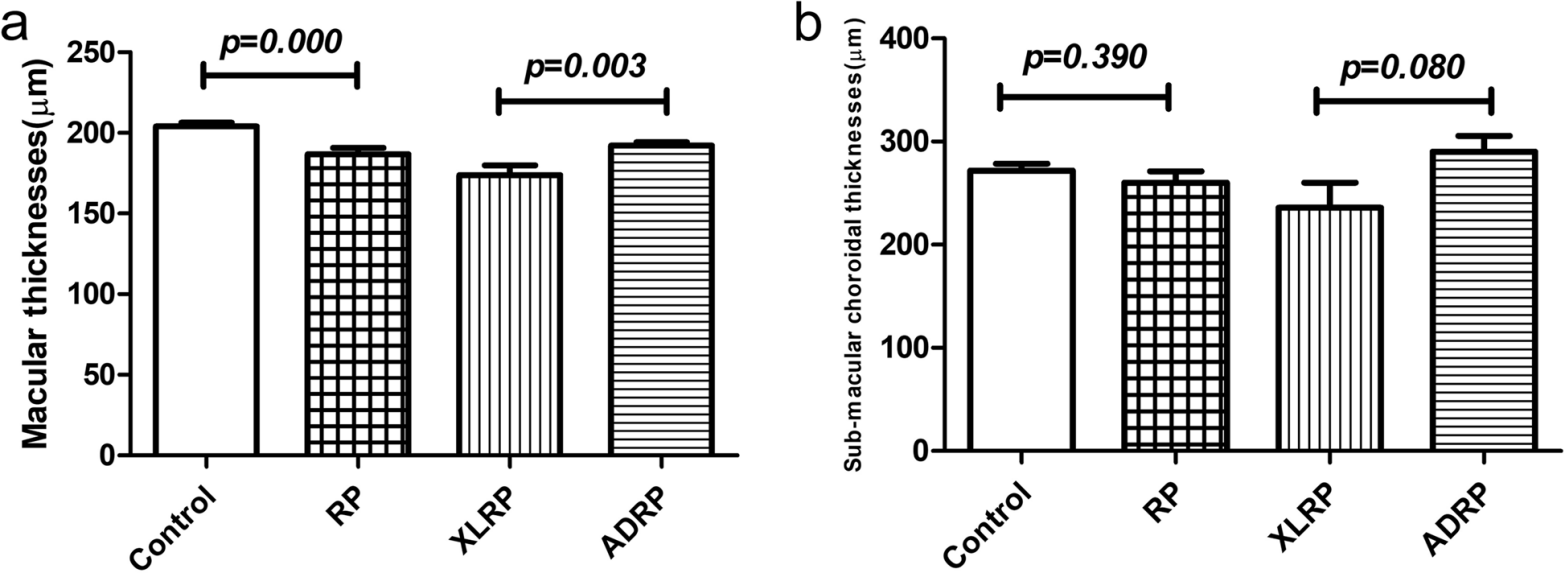
Macular thicknesses and Sub-macular choroidal thicknesses (CT) in control group and retinitis pigmentosa (RP) group and different genetic subgroups. **a** There were significant differences in the macular thicknesses between the control group and the RP group (*p* = 0.000). The foveal RT of X-linked retinitis pigmentosa (XLRP) patients was less than that of autosomal dominant retinitis pigmentosa (ADRP) patients (*p* = 0.003). **b** There was no significant thinning on sub-macular CT between the control group and the RP group, and between XLRP and ADRP

There were significant differences in the RT between the RP group and the control group (*p* = 0.000) at all of the measured points (Table 2). In the RP group, the mean CT 3000 μm to the temporal side of the fovea was 243.76 ± 60.82 μm, while the corresponding measurement in the control group was 275.23 ± 40.92 μm; this difference was significant (*p* < 0.05; shown in Table 3). There was no significant thinning in the other locations we studied. We found that the CT measurements of RP children showed a more severe reduction on the temporal side. Macular thicknesses and sub-macular CT of the RP group and the control group were presented in Fig.3.

**Table 2:** Retinal thicknesses of each group

Retinal thicknesses (μm)	Control (***n*** = 28, 56 eyes)	RP (***n*** = 32, 64 eyes)	***P*** value	RP (***n*** = 17, 34 eyes)		
				XLRP (*n* = 7, 14 eyes)	ADRP (*n* = 10, 20 eyes)	*P* value
T2	250.89 ± 12.00	200.75 ± 40.23	0.000	199.57 ± 43.03	211.80 ± 29.78	0.344
T1	316.00 ± 14.03	270.09 ± 36.23	0.000	260.92 ± 40.22	281.70 ± 23.73	0.067
M	203.14 ± 15.44	186.88 ± 30.46	0.000	173.85 ± 22.87	192.20 ± 9.70	0.003^*^
N1	327.25 ± 14.45	284.45 ± 43.12	0.000	270.14 ± 48.77	295.45 ± 24.17	0.054
N2	281.89 ± 25.08	235.06 ± 40.12	0,000	228.92 ± 29.77	249.50 ± 34.47	0.080

*Abbreviations*: *M* Foveal thickness, *N1* Thickness 1000 μm nasal to the fovea, *N2* Thickness 3000 μm nasal to the fovea, *T1* Thickness 1000 μm temporal to the fovea, *T2* Thickness 3000 μm temporal to the fovea. *XLRP* X-linked RP, *ADRP* Autosomal dominant RP

**Table 3:** Choroidal thicknesses of each group

Choroid thicknesses (μm)	Control (***n*** = 28, 56 eyes)	RP (***n*** = 32, 64 eyes)	***P*** Value	RP with confirmed gene mutations		
				XLRP (*n* = 7, 14 eyes)	ADRP (*n* = 10, 20 eyes)	*P* value
T2	275.23 ± 40.92	243.76 ± 60.82	0.001^*^	211.21 ± 69.41	274.45 ± 57.91	0.007^*^
T1	284.19 ± 43.93	262.61 ± 78.75	0.072	222.78 ± 94.02	279.05 ± 69.13	0.053
M	271.69 ± 50.29	260.17 ± 88.00	0.390	235.35 ± 108.05	290.15 ± 68.87	0.080
N1	238.94 ± 52.22	231.87 ± 90.79	0.609	203.78 ± 98.34	249.00 ± 70.61	0.128
N2	145.12 ± 43.09	145.53 ± 79.27	0.973	121.57 ± 72.73	149.30 ± 63.05	0.245

**Fig. 4:**
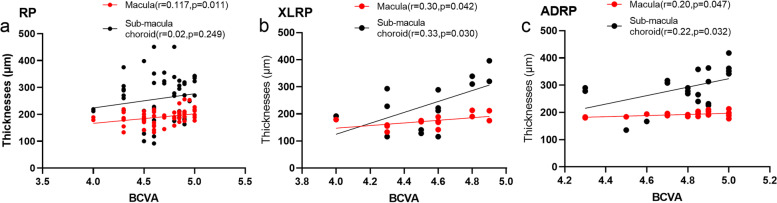
Best-corrected visual acuity (BCVA) and macular or sub-macula choroidal thicknesses. **a** There were positive correlations between BCVA and macular thicknesses in retinitis pigmentosa (RP) group while no correlations with sub-macula choroidal thicknesses. **b** There were positive correlations between BCVA and macular thicknesses, and between BCVA and sub-macula choroidal thicknesses in X-linked RP group. **c** There were positive correlations between BCVA and macular thicknesses, and between BCVA and sub-macula choroidal thicknesses in autosomal dominant RP group

The mean RT of the XLRP and ADRP group are presented in Table 2. There were significant differences in the foveal RT between the two groups (*p* = 0.003). The foveal RT of XLRP patients was less than that of ADRP patients (Fig.3), but there was no significant difference at 1000 μm or 3000 μm from the fovea. We found that retinal degeneration at the macula was more obvious in children with XLRP than in children with ADRP. The macular thicknesses were positively correlated with BCVA in XLRP and ADRP respectively (Fig.4). The visual acuity of the XLRP group was worse than that of the ADRP group (Table 2), which also indicates that XLRP leads to seriously damage.

**Fig. 5:**
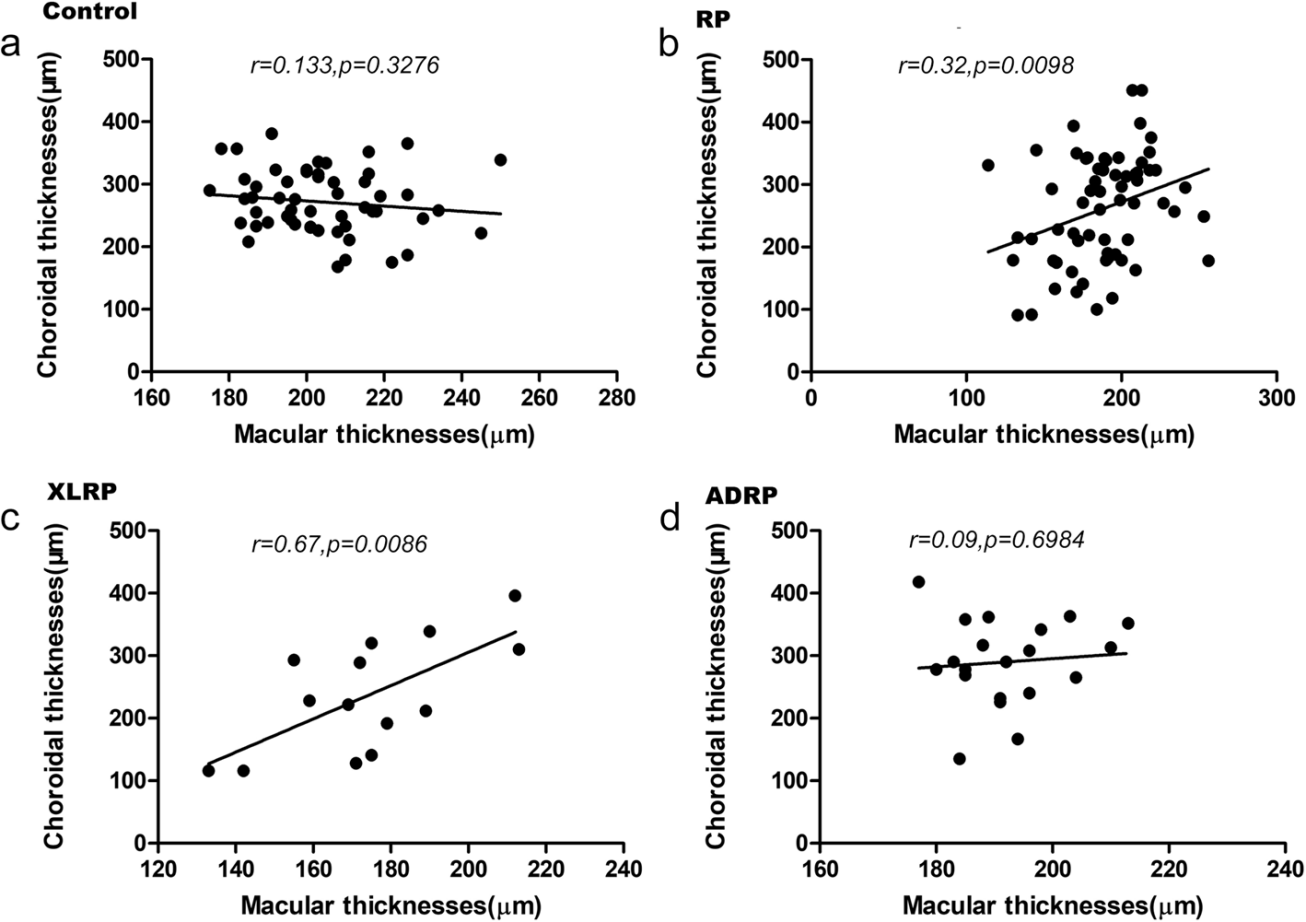
Correlations between macular thicknesses and sub-macular choroidal thicknesses. **a** There were no correlations between macular thicknesses and sub-macular choroidal thicknesses in control group. **b** and **c** There were positive correlations between macular thicknesses and sub-macular choroidal thicknesses in retinitis pigmentosa group and X-linked retinitis pigmentosa group. **d** There was no correlations between macular thicknesses and sub-macular choroidal thicknesses in autosomal dominant retinitis pigmentosa group

**Fig. 6:**
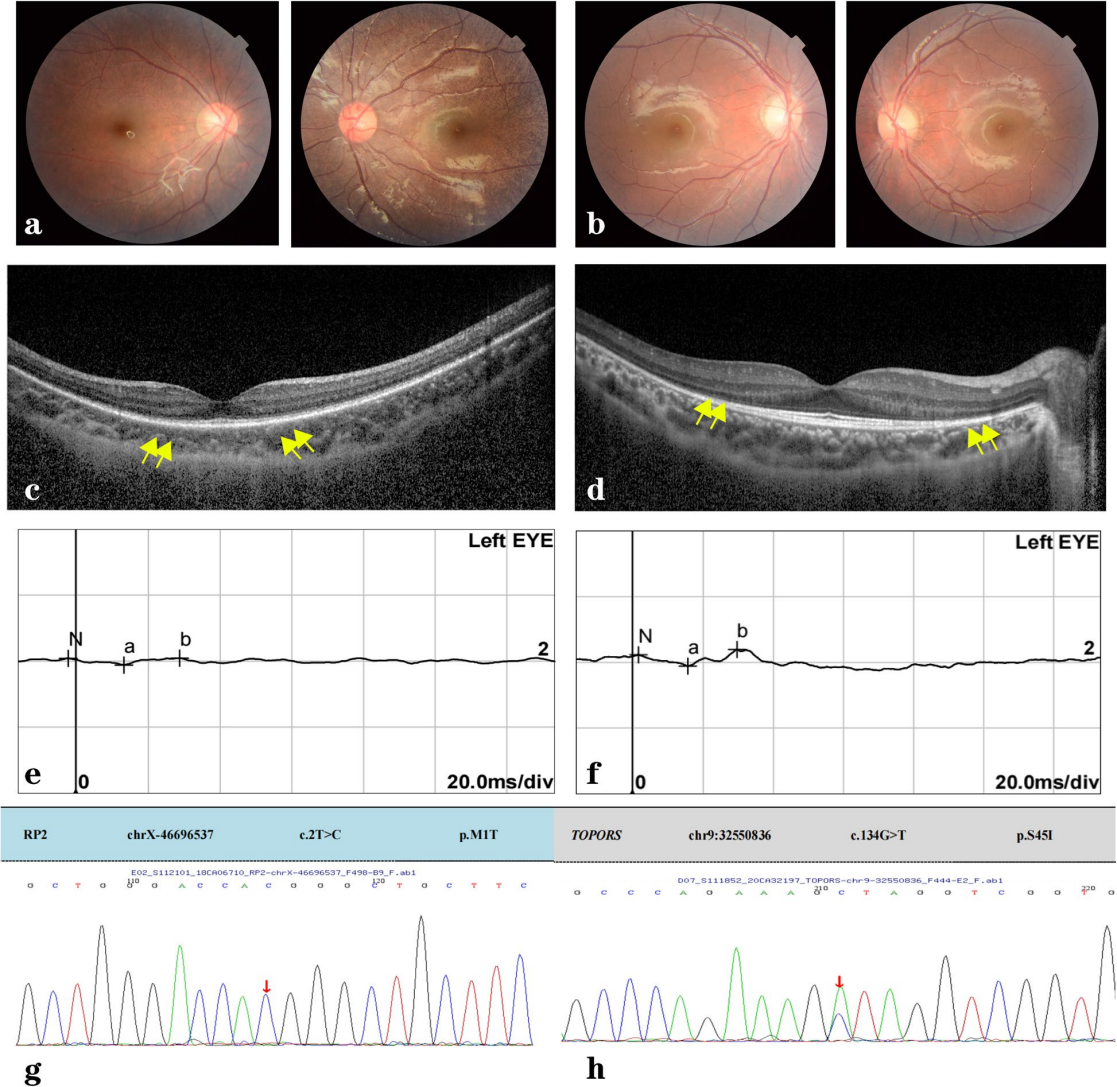
Ocular fundus and OCT images, ERGs and gene assays of 2 patients with X-linked retinitis pigmentosa (XLRP) and autosomal dominant retinitis pigmentosa (ADRP). Case 1 XLRP (**a**, **c**, **e**, **g**), male, 6 years old, night blindness for 2 years. Best-corrected visual acuity (BCVA): R: 0.6, L: 0.7. His uncle, mother and grandmother had RP. Case 2 ADRP (**b**, **d**, **f**, **h**), male, 7 years old, mild night blindness, with family history. BCVA: R:1.0, L:1.0. His mother and grandmother had RP. Case 1 (XLRP) showed more pigment on the ocular fundus image, a thinner photoreceptor layer on OCT imaging, more obvious ellipsoid band disappearance (area between yellow arrows) and a smaller electrophysiology signal than case 2 (ADRP), as well as more serious functional loss

The mean CT of the XLRP and ADRP group are presented in Table 3. The CT of the XLRP group 3000 μm to the temporal side of the fovea was smaller than that of the ADRP group, while there was no significant thinning in the other locations we studied (*p* = 0.007). We found that the CT measurements of the XLRP children showed a more severe reduction on the temporal side than those of ADRP children. The temporal thinning of the choroid was more pronounced in children with RP and XLRP than in the control and ADRP groups, respectively. There were positive correlations between macular thicknesses and sub-choroidal thicknesses in RP group (Fig.5). Some clinical data from 2 patients with XLRP and ADRP are shown in Fig. 6.

## Discussion

Our results suggest that the thickness of the retina of RP children is smaller than that of the control group and that the RT at the fovea is smaller in XLRP children than in ADRP children. There are diurnal variations in the retina and choroid measurements, so we carried out our OCT examinations in the afternoon [9]. The decrease in the RT with the progression of RP is due to progressive degeneration of the rod and cone cells; the typical early changes in RP are represented by shortening of the outer segments of photoreceptors, with gradual degeneration of the cones [10]. In a study by Yildirim, M.A., the RT of RP patients was significantly lower than that of normal subjects [11]. The disease affects the entire retina but shows regional variation in the severity of degeneration [12]. Thus, the RT values obtained in our study indicated that the severity of damage near the macula was greater in the XLRP group than in the ADRP group. The reduction in the thickness in the macular area is more serious in children with an XLRP genotype than in those with an ADRP genotype. Our findings also provided evidence that the changes in thicknesses may be representative of the greater severity of XLRP versus ADRP in the early stage.

Thinning on the temporal side of the choroid was more obvious in RP than in healthy children and in XLRP than in ADRP children. This choroidal thickening is different from that in children with simple myopia, for whom it begins in central foveal regions [13]. The choroid provides nutrition to outer retinal structures and plays a significant role in the pathophysiology of RP [14]. The CT is significantly lower in RP patients than in normal controls [15, 16]. As the sub-choroidal thicknesses thinning, the macular thicknesses become smaller in RP. Histopathological studies have confirmed that poor choroidal blood flow leads to thinning of the choroid, which also leads to photoreceptor damage. The loss of photoreceptors results in decreased oxygen demand and a consequent reduction in the blood supply. The quantification of CT could reflect changes in ocular vascular perfusion [17]. Our results suggest that the temporal flow is more likely to be affected. Therefore, retinal degeneration may stimulate choroidal atrophy, and conversely, choroidal changes may worsen the degeneration of the outer retina. This may also explain why XLRP patients develop symptoms earlier and have more severe impairment than ADRP patients.

The severity of the disease is closely related to its heterogeneous genetic basis. The severe forms of RP are within the category of X-linked retinitis pigmentosa. Patients are characterized by a severe phenotype even in the early stages of disease development [18], with earlier onset of night blindness, more rapid constriction of visual fields and, eventually, more severe loss of central acuity than in autosomal dominant retinitis pigmentosa [19]. Most affected males exhibit early-onset symptoms, with night blindness in the first decade, a reduction in the visual field in the second decade, and rapid progression towards blindness (worse than approximately 20/200) by 40 years of age [20]. The majority of mutations that cause XLRP lead to abnormal protein distribution in the cell [21]. As the disease progresses, it affects the function of tubulin and ciliary structures and causes increasing damage to structures and function [22]. A number of retinal proteins are required for cilia-dependent outer segment transport, generation or maintenance [23]. The abnormality of the connecting cilia could result in decreased protein transport from the inner to the outer photoreceptor [24].

Two genotypes of XLRP are common in the clinic: RPGR and RP2. RPGR is characterized by conserved guanine nucleotide exchange factors. The protein localizes to the outer segment of photoreceptors. RPGR defects can occur at multiple steps in material transport towards the distal outer segment [23]. Due to the importance of these pathways in photoreceptor development and survival, mutations in RPGR might disrupt the interactions and lead to retinal degeneration. Mutations in RP2 cause the second most frequent form of XLRP [21]. The gene products show homology with human cofactor C, which acts as a GTPase-activating protein in the final step of beta-tubulin folding. RP2 also interacts with ADP-ribosylation factor-like 3 (ARL3), which acts as a microtubule-associated small GTP-binding protein that localizes to the sensory cilia of photoreceptors [23]. Mutations in RP2 lead to the accumulation of incorrectly folded photoreceptor- or neuron-specific tubulin isoforms, resulting in the progressive degeneration of the retina.

ADRP is a relatively mild form of RP. The gene mutations associated with ADRP can result in altered protein activity, endoplasmic reticulum dysfunction due to misfolded mutant protein molecules, mistrafficking and mislocalization of the mutant protein, and even adverse effects on the proper functioning of the wild-type protein, thus affecting the homeostasis of cells and ultimately resulting in their death [25]. ADRP includes 30 genotypes, such as RHO mutations. RHO mutations are associated with two phenomena: interference with the function of normal rhodopsin and intrinsic toxicity of the mutant protein. Most of the mutations lead to opsin misfolding and instability, leading to a relatively mild disease phenotype [26].

In conclusion, the choroid in RP children becomes thinner preferentially on the temporal side of the macula, and thinning of the retina is relatively extensive. Children with RP have strong clinical and genetic heterogeneity. The XLRP children demonstrated greater RT reduction at the fovea and greater CT reduction at the temporal side of the macula than the ADRP children. Our findings also provided evidence that the changes in thicknesses may be indicative of the greater severity of XLRP versus ADRP in the early stage.

This study has some limitations. The retinal and choroidal thicknesses were measured manually, which may create a potential bias. The study sample size was small, and larger studies should be conducted. Axial length can interfere with accurate retinal and choroidal thickness measurements, and our future research should be more comprehensive. As this was a retrospective study, the data are prone to bias. Additionally, further studies should investigate whether the refractive state of children with XLRP and children with ADRP affects retinal thickness. We should also perform more refined genetic data analyses, which may reveal other factors contributing to the diagnosis and prognosis of RP. Further studies might enhance our clinical evaluation of such patients.

## Data Availability

The datasets generated and/or analysed during the current study are not publicly available because the follow-up research, including treatment and effect on these patients, is still ongoing, but they are available from the corresponding author upon reasonable request.
